# Application of artificial intelligence models in the identification of severe scrub typhus

**DOI:** 10.3389/frai.2026.1842584

**Published:** 2026-07-22

**Authors:** Chunyu Chen, Xiangling Liu, Ce Yang, Chuncheng Ma, Qingxia Zhou, Junying Zeng, Yinan Chen, Peipei Ding, Ran Li, Wencong Liang, Zhiyong Hong, Chuanbo Qin, Gang He

**Affiliations:** 1 Department of Infectious Diseases, Jiangmen Central Hospital, Jiangmen, China; 2 Endoscopy Center of Jiangmen Central Hospital School of Electronic and Information Engineering, Wuyi University, Jiangmen, China; 3 Infection Department of Wuyi Traditional Chinese Medicine Hospital, Jiangmen, China; 4 Department of Infectious Diseases, Xinhui People’s Hospital, Jiangmen, China; 5 Department of Oral and Maxillofacial Surgery, Jiangmen Central Hospital, Jiangmen, China; 6 Endoscopy Center of Jiangmen Central Hospital, Jiangmen, China

**Keywords:** artificial intelligence, critical condition, machine model, predictive model, scrub typhus

## Abstract

This retrospective study enrolled 492 patients with scrub typhus in Jiangmen from 2013 to 2025. Clinical and laboratory data were analyzed using univariate logistic regression and LASSO regression to identify risk factors for severe illness. Seven machine-learning models, including logistic regression, support vector machine, random forest, XGBoost, Naive Bayes, k-nearest neighbor, and decision tree, were constructed and externally validated. Variable importance was ranked using SHAP analysis. Mechanical ventilation represented the strongest predictor of severe disease, indicating that respiratory failure is the core indicator of disease progression. Elevated bilirubin, prolonged coagulation time, and thrombocytopenia were also closely associated with severe scrub typhus. XGBoost achieved the best predictive performance, with AUC values of 0.888 and 0.928 in the training and validation cohorts, respectively. Owing to limited positive cases, mortality prediction yielded lower AUCs (0.856 and 0.814). These findings demonstrate that artificial intelligence models can effectively stratify the severity of scrub typhus and present favorable potential in prognostic assessment.

## Introduction

Scrub typhus is an acute endemic infectious disease caused by *Orientia tsutsugamushi* and transmitted via the bites of trombiculid mite larvae. It is highly prevalent throughout the Asia-Pacific region, particularly in Southeast Asia. Its endemic coverage spans eastward to Japan, westward to Afghanistan, southeastward to northern Australia, and northeastward to the coastal Russian Far East, forming the well-recognized “scrub typhus” (Luce-Fedrow et al., 2018; Cosson et al., 2015; Maina et al., 2011). In China, following the reinstatement of the national direct reporting system for notifiable infectious diseases in 2006, the incidence of scrub typhus has been steadily rising, accompanied by a progressive expansion of its epidemic geographical coverage (Qin et al., 2025; Yue et al., 2020). As of now, except for Shanghai and the Ningxia Hui Autonomous Region, a total of 30 provinces, autonomous regions, and municipalities nationwide have reported indigenous cases, with Yunnan, Guangdong, and Fujian being high-incidence areas (Yue et al., 2020; Musa et al., 2021; Peng et al., 2025).

Following infection with *Orientia tsutsugamushi*, the incubation period typically lasts 5 to 14 days. Typical clinical manifestations consist of fever, rash, pathognomonic eschars or ulcers, headache, myalgia, cough, lymphadenopathy and hepatosplenomegaly (Xu et al., 2017; Alam et al., 2022; Won et al., 2023). Severe cases are prone to multisystem complications, including toxic hepatitis, acute kidney injury, pneumonia, acute respiratory distress syndrome (ARDS), myocarditis, meningitis, encephalitis, septic shock and multiple organ failure (Devasagayam et al., 2021; Jamil et al., 2014; Tiwari et al., 2015). In the absence of timely and effective treatment, the case fatality rate can reach 14.3% to 70% (Taylor et al., 2015; Wang et al., 2024).

Guangdong Province represents a historically high-burden region of scrub typhus, with a marked upward trend in notified incidence over recent years (Wei et al., 2021; Fan et al., 2025). A proportion of patients may progress to severe scrub typhus as a result of delayed diagnosis and clinical intervention, which underscores the clinical value of early recognition of severe cases. Alongside the rapid advancement of artificial intelligence, machine learning algorithms have been extensively utilized in disease prediction and auxiliary diagnosis, benefiting from their superior performance in multi-dimensional data analysis, pattern identification and predictive visualization (Rajkomar et al., 2019; Greener et al., 2022; Huang et al., 2025).

Against this epidemiological backdrop, this study aimed to conduct early prediction of disease severity and mortality risk among patients with scrub typhus. Clinical data of scrub typhus patients hospitalized at Jiangmen Central Hospital over the past decade were retrospectively retrieved to develop multiple machine learning predictive models. Independent datasets from additional medical centers were used as external validation cohorts to rigorously evaluate model generalizability, thereby providing robust evidence for formulating precise clinical prevention and management strategies.

### Materials and method

### Data source

The research data were collected from three medical centers: Jiangmen Central Hospital (2013–2025, 394 cases), Wuyi Hospital of Traditional Chinese Medicine (2015–2025, 93 cases), and Xinhui People’s Hospital (2020–2025, 8 cases). We extracted comprehensive clinical data of these scrub typhus patients from the hospital electronic medical record system, covering demographic characteristics, clinical manifestations, laboratory indicators, treatment progress, and prognosis.

*Inclusion criteria*

Clinical diagnosis of ST was made for patients who: 1. Had a recent history (within 3 weeks) of outdoor exposure in endemic regions, presenting with an acute high fever accompanied by typical symptoms such as an eschar or ulcer, rash, swollen lymphnodes, and other characteristic clinical signs. 2. Showed at least one positive laboratory result, which could include specific IgM antibodies, a fourfold rise in serum antibody levels (detected through the Weil-Felix test), or pathogen identification using polymerase chain reaction (PCR) (Jiang et al., 2025).

*Exclusion criteria*

1. History of Malignant Tumors、congenital heart disease, or immunodeficiency. 2. Hepatorenal Insufficiency. 3. Significant Deficiencies in Clinical Information.

Severe case criteria: (Li et al., 1952; Roychowdhury et al., 2022).

Severe complications refer to the occurrence of any of the following new problems or conditions in patients: 1. Central nervous system complications: altered mental status, seizures, intracranial hemorrhage, and/or cerebral infarction. 2. Respiratory complications: both of the following conditions are met: imaging shows bilateral pulmonary infiltrates; and at least one of the following is met: oxygenation index (PaO₂/FiO₂) ≤ 250, respiratory rate ≥ 30 breaths/min, or mechanical ventilation is required. 3. Cardiac complications: any of myocarditis, malignant arrhythmia, or ischemic heart disease. 4. Renal complications: defined as a serum creatinine level ≥ 2 mg/dL, or worsening of pre-existing renal injury requiring acute renal replacement therapy (e.g., dialysis). 5. Septic shock: requiring intravenous vasopressors for more than 1 h to maintain a systolic blood pressure > 90 mmHg. 6. APACHE II scoring criteria: APACHE II score ≥ 10 points upon initial admission (within 24 h) defines a severe case.

### Construction and validation of predictive models

#### Feature selection

Feature selection is an indispensable step in machine learning modeling. It aims to screen variables most relevant to the prediction target, so as to reduce data dimensionality, improve training efficiency and enhance model generalization ability.

To identify clinical indicators significantly correlated with severe deterioration and mortality of scrub typhus, L1 regularization was adopted for continuous variables in this study. Variable selection was realized through coefficient sparsification. This strategy can constrain overfitting while preserving features with the highest predictive value for clinical outcomes, thereby obtaining interpretable and robust candidate indicators for subsequent analysis and clinical application. Repeated cross-validation was performed to evaluate the stability and uncertainty of selected features, and the selection frequency of each variable was recorded. Only variables with high selection frequency were incorporated into the final candidate list. The retained variables were further refitted via non-regularized logistic regression to calculate calibrated regression coefficients and odds ratios (OR) with 95% confidence intervals. Permutation test and bootstrap method were used to determine statistical significance and confidence intervals. Model performance was assessed based on the area under receiver operating characteristic curve (AUC), sensitivity, specificity and other quantitative metrics.

#### Model construction

Data from Jiangmen Central Hospital was defined as the training set, and data from Wuyi Traditional Chinese Medicine Hospital and Xinhui People’s Hospital were used as the external validation set. All model construction and parameter learning were completed based on the training set, whereas the external validation set was only applied to independently assess the generalization capacity of the final model.

In terms of methodology, seven models were adopted in this study, including Logistic Regression (LR), Support Vector Machine (SVM), Random Forest (RF), eXtreme Gradient Boosting (XGBoost), Naïve Bayes (NB), K-Nearest Neighbors (KNN) and Decision Tree. These models cover linear and highly nonlinear algorithms, parametric and non-parametric approaches, as well as basic baseline models and high-performance ensemble algorithms. Each model has unique modeling properties. As a highly interpretable and cost-effective baseline model, logistic regression can quantify the action direction and influence intensity of clinical and laboratory indicators on the risk of severe illness. Supported by kernel functions, support vector machine can fit complex classification boundaries under small-to-medium sample sizes and high-dimensional feature conditions. Random forest and XGBoost both belong to tree-based ensemble models. Random forest shows strong stability when processing heterogeneous clinical data and enables convenient evaluation of feature importance. With integrated gradient boosting and regularization strategies, XGBoost usually delivers superior predictive performance on structured tabular data. Naïve Bayes is applicable to high-dimensional sparse data and samples with prior information, and can quickly generate baseline probabilistic classification results. As a non-parametric local modeling method, K-Nearest Neighbors can identify similar patient clusters and reflect local data distribution patterns.

The included models cover linear models, kernel methods, distance-based discriminant models, decision tree models, ensemble learning models and probabilistic classification models, allowing comprehensive comparison of predictive performance across different modeling strategies. Stratified K-fold cross-validation was conducted within the training set for hyperparameter optimization. GridSearchCV was utilized to traverse the predefined parameter ranges, and the area under receiver operating characteristic curve (AUC) was regarded as the core optimization indicator. Cross-validation was entirely implemented in the training set. Model threshold determination and preliminary performance evaluation were finished based on internal validation results of the training set. The external validation set was excluded from model training, parameter tuning and threshold selection, and only served for independent generalization verification. The combination of diverse models helps excavate potential risk signals from varying modeling assumptions and model complexity. It also provides a foundation for subsequent probability calibration and model interpretability analysis such as SHAP value analysis and local interpretation algorithms, so as to balance model predictive performance and clinical interpretability. The hyperparameter-optimized XGBoost model was finally selected, and SHAP attribution analysis was carried out based on feature subsets screened by L1 regularization. Death and severe adverse outcomes were originally coded as 0 in raw data. To unify the event judgment standard and guarantee that SHAP value interpretation conforms to clinical practical significance, outcome labels were recoded before analysis, and adverse outcomes were defined as positive events.

#### Model performance evaluation and validation

Model performance was evaluated on the independent test set. Severe cases were defined as the positive class, while mild cases were classified as the negative class. Based on this definition, multiple evaluation metrics were calculated, including sensitivity (recall), precision, specificity, accuracy and F1-score. Moreover, receiver operating characteristic (ROC) curves were plotted, and the area under the curve (AUC) was calculated for each model. Models achieving higher values of these metrics were considered to have better predictive performance. Decision curves and calibration curves of each model were generated in both internal and external validation sets to evaluate the consistency between model-predicted probabilities and the actual observed incidence of clinical outcomes. In addition, the Brier score was calculated and reported as a quantitative indicator reflecting the overall calibration performance and probabilistic prediction error of the established models. This approach enabled a comprehensive assessment of the clinical reliability of model predictive probabilities.

#### Statistical analysis

All statistical analyses were performed using Python. Variables with a missing data rate higher than 20% and obvious outliers were excluded first, and the remaining missing data were imputed using multiple imputation. Continuous variables conforming to a normal distribution were described as mean ± standard deviation (x̄ ± s), and independent samples *t*-test was used for intergroup comparisons. Continuous variables not conforming to a normal distribution were expressed as median (interquartile range), and Mann–Whitney U test was used for intergroup comparisons. Categorical variables were expressed as frequency and percentage [*n* (%)]. The significance level for hypothesis testing was set at *p* < 0.05.

#### Data preprocessing and classification threshold determination

All continuous variables were first subjected to missing value imputation using median values calculated from the training set. Standardization was subsequently implemented with Standard Scaler. The imputation model and standardization parameters were exclusively fitted on the training dataset. Internal and external validation sets were merely transformed based on parameters acquired from the training set, so as to avoid data leakage. Outliers of continuous variables were determined by the interquartile range (IQR) method. Observations lower than Q1 − 1.5 × IQR or higher than Q3 + 1.5 × IQR were defined as outliers. To prevent selection bias induced by subjective deletion, arbitrary elimination of outliers was not conducted in this study. Alternatively, all outliers were statistically marked and summarized. The outlier threshold range and quantity of each continuous variable were listed.

The optimal cut-off threshold of each model was determined in the internal validation set based on the Youden index, which maximized the value of sensitivity+specificity−1. The obtained threshold was fixed and directly applied to the external validation set. The external validation set was only used to evaluate model generalizability and not involved in threshold re-selection, so as to prevent optimistic bias in external validation outcomes.

#### Class imbalance handling

Since outcomes of death and severe illness are relatively rare events in real-world clinical cohorts, the dataset in this study presented obvious outcome class imbalance. Without effective correction, machine learning models tend to be biased toward the majority class during training, which leads to overestimation of overall accuracy and reduced capability to identify rare clinical events. Therefore, we adopted a class imbalance handling strategy based on cost-sensitive learning in the model development phase. Specifically, higher weights were assigned to minority outcome classes throughout model optimization, enabling the models to attach greater importance to the misclassification risk of clinically critical endpoints including death and severe cases during training.

For models supporting class weight settings, class weights were automatically adjusted according to the sample frequency of different outcome classes in the training set to grant larger weights to minority classes. For ensemble tree models, weight correction was applied in each iteration of model training and subsample generation, so as to reduce the dominance of majority classes over model splitting rules and predictive results. For XGBoost, we regulated the loss contribution of minority classes in the gradient boosting process to improve the model’s discriminative performance for low-incidence outcomes during iterative optimization. In the original dataset, death and severe cases were coded as 0. These endpoints were redefined as positive events only for model optimization purposes within the internal training process of XGBoost. This recoding operation did not change the original definition of outcomes or relevant statistical interpretations.

**Figure 1:**
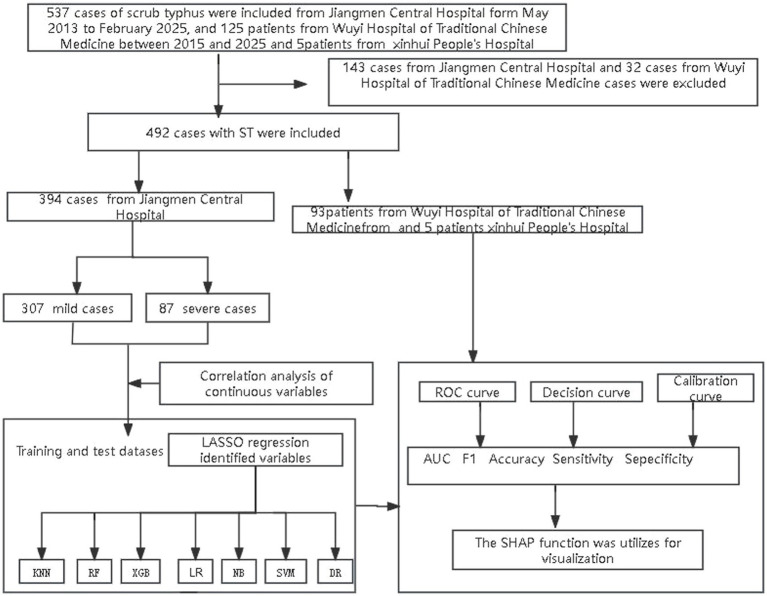
Technical Roadmap.

In the primary analysis, we mainly adopted the built-in weight correction functions of the models, rather than oversampling or undersampling methods. This design aimed to fully retain the natural outcome distribution of the real clinical cohort and avoid extra distribution shift introduced by artificial alteration of sample structures. Detailed parameter settings for class imbalance handling of each model are provided (see Figure 1).

## Results

### Demographic data and clinical characteristics of enrolled cases

From February 2013 to February 2025, a total of 537 patients were screened, of whom 143 were excluded due to not meeting the inclusion criteria, leaving 394 patients to be included in this study as the experimental group. We obtained various characteristics of scrub typhus patients, but in order to reduce the influence of human subjectivity, we selected 56 objective characteristics.

Among them, there were 87 severe cases in our hospital, with 36 deaths; 20 severe cases in Wuyi Hospital of Traditional Chinese Medicine, with 7 deaths; and 5 severe cases in Xinhui People’s Hospital, with 3 deaths. The baseline characteristics of the patients are shown in Table 1. Comparison of baseline characteristics between the training group and the external validation group (98 cases) showed no significant difference in age distribution between the two groups (median age of the training group was 62 years, IQR: 53–69; median age of the validation group was 63.5 years, IQR: 55–70; *p* = 0.212). Males slightly predominated in the training group (51.53%). The median treatment duration of doxycycline or chloramphenicol was 7 days (IQR: 6–10). A total of 42 cases (10.7%) were admitted to the ICU, 43 cases (10.9%) required mechanical ventilation, and 34 cases (8.6%) died. Among the included laboratory characteristics and disease-related variables, 23 indicators including white blood cell (WBC), platelet (PLT), procalcitonin (PCT), C-reactive protein (CRP), prothrombin time (PT), activated partial thromboplastin time (APTT), alanine aminotransferase (ALT), aspartate aminotransferase (AST) and creatinine (Cr), as well as 6 disease variables, showed statistically significant differences between groups (*p* < 0.05), while the remaining variables showed no significant differences between groups (*p* > 0.05).

**Table 1:** Baseline features of the training set and external validation set.

Variable	Total (*n* = 394)	Total (*n* = 98)	*p*
Patient characteristics			
Sex (male) [*n*(%)]			0.03
No	192 (48.73)	47 (47.96)	
Yes	202 (51.53)	51 (52.04)	
Age (median[IQR], years)	62 (53, 69)	63.5 (55, 70)	0.212
BMI (median[IQR])	22.3 (19.9, 24.68)	23.1 (21.05, 24.5)	0.344
Treat time (median[IQR], d)	7.0 (6.0, 10.0)	7.0 (4.0, 10.0)	0.233
Smoke [*n*(%)]	88 (22.3)	28 (28.6)	1
Drink [*n*(%)]	24 (6.1)	10 (10.2)	0.182
Ventilator [*n*(%)]	43 (10.9)	20 (20.4)	0.88
ICU stay [*n*(%)]	42 (10.7)	20 (20.4)	<0.001
Severe Scrub Typhus [*n* (%)]	87 (22.08)	25 (25.5)	
Death [*n*(%)]	36 (9.1)	10 (10.2)	
基础疾病			
Hypertension [*n*(%)]	61 (15.5)	29 (29.6)	0.263
Diabetes [*n*(%)]	44 (11.2)	18 (18.4)	1
Hepatic insufficiency	185 (47.0)	41 (41.9)	0.019
Thrombocytopenia [*n*(%)]	35 (8.9)	9 (9.2)	0.224
Renal insufficiency [*n*(%)]	74 (18.8)	14 (14.3)	0.210
Sepsis [*n*(%)]	31 (7.9)	13 (13.3)	<0.001
MODS [*n*(%)]	56 (14.2)	15 (15.3)	0.167
Coronary heart disease [*n*(%)]	16 (4.1)	4 (4.1)	0.660
Hepatitis B [*n*(%)]	19 (4.8)	6 (6.1)	0.707
Respiratory failure [*n*(%)]	25 (6.4)	7 (7.1)	<0.001
Cardiac insufficiency [*n*(%)]	39 (9.9)	10 (10.2)	<0.001
Atrial fibrilation [*n*(%)]	11 (2.8)	2 (2.04)	0.213
Pneumonia [*n*(%)]	139 (35.3)	47 (47.96)	0.219
Tuberculosis [*n*(%)]	6 (1.5)	1 (1.02)	1
Syphilis [*n*(%)]	3 (0.8)	1 (1.02)	<0.001
Laboratory findings			
WBC (median [IQR], ×10^9^/L)	8.18 (5.79, 10.74)	7.78 (5.69, 9.78)	0.003
Neu(%) (median [IQR], %)	70.03 (56.9, 80.7)	80.75 (71.17, 87.55)	0.027
LYM (median [IQR], %)	22.43 (12.8, 34.2)	13.75 (7.83, 21.15)	0.042
MON(%) (median [IQR],%)	5.9 (3.48, 9.14)	5.3 (3.2, 7.9)	0.020
RBC (median [IQR], ×10^9^/L)	4.16 (3.71, 4.55)	4.3 (3.94, 4.67)	0.375
HGB (median [IQR], g/L)	120 (107, 131.3)	126 (112, 134)	0.089
PLT (median [IQR], ×10^9^/L)	120 (70, 182.5)	113 (80.2, 160.7)	<0.001
PCT (median [IQR], ng/ml)	0.76 (0.33, 1.96)	0.72 (0.38, 1.82)	<0.001
CRP (median [IQR], mg/L)	87.7 (41.5, 137.2)	80.8 (48.94, 131.97)	0.024
PT (median [IQR], s)	12.4 (11.6, 13.4)	12.4 (11.6, 13.07)	<0.001
INR (median [IQR])	1.04 (0.97, 1.13)	1.07 (1.01, 1.13)	<0.001
PTA (median [IQR], %)	88.3 (76.35, 102)	83.65 (73.1, 92.55)	0.014
Fib (median [IQR], g/L)	2.72 (1.97, 3.51)	3.34 (2.74, 4.21)	0.012
APTT (median [IQR], s)	31.9 (28.0, 37.3)	30.7 (28.9, 35.8)	<0.001
DDi (median [IQR], mg/L)	4.77 (2.22, 11.1)	6.73 (3.46, 13.55)	<0.001
ALT (median [IQR], IU/L)	73.0 (46.0, 122.2)	70.8 (45.0, 112.25)	0.245
AST (median [IQR], IU/L)	81.8 (49.8, 138.5)	93.5 (58.25, 137.43)	<0.001
ALP (median [IQR], IU/L)	111.0 (75, 171)	105.0 (75.0, 164.5)	0.002
TBIL (median [IQR], umol/L)	10.0 (7.35, 15.6)	11.65 (8.83, 16.6)	<0.001
DBIL (median[IQR], umol/L)	5.0 (3.2, 8.45)	6.2 (3.95, 9.25)	<0.001
ALB (median [IQR], g/L)	29.6 (26.1, 33.95)	33.6 (30.3, 37.3)	0.005
TG (median [IQR], mmol/L)	2.26 (1.55, 3.39)	2.32 (1.54, 3.43)	0.003
TC (median [IQR], mmol/L)	3.4 (2.7, 4.08)	3.37 (2.83, 4.12)	0.071
LDL-C (median [IQR], mmol/L)	1.79 (1.19, 2.48)	2.06 (1.27, 2.53)	0.010
Urea (median [IQR], mmol/l)	5.01 (3.42, 8.48)	5.26 (3.96, 8.3)	<0.001
Cr (median [IQR], umol/l)	79.1 (64.0, 102.7)	84.5 (67.25, 111.75)	<0.001
GFR (median [IQR], ml/min/1.73 m^2^)	82.6 (57.7, 96.5)	72.0 (51.1, 90.4)	<0.001
CKMB (median [IQR])	1.92 (0.81, 4.79)	22.3 (13.65, 128.0)	0.102
TnIH (median [IQR])	0.03 (0.01, 0.08)	0.02 (0.01, 0.04)	0.888
NT-ProBNP	1,586 (640.4483)	119.8 (46.4, 1653.0)	0.314
Na (median [IQR], mmol/L)	136.0 (132.6, 138.7)	133.9 (130.8, 137.1)	0.199
K (median [IQR], mmol/L)	3.6 (3.3, 3.97)	3.73 (3.36, 3.97)	0.456
Cl (median [IQR], mmol/L)	100 (96, 103)	98.4 (94.7, 102.3)	0.116

WBC, white blood cell; Hb, hemoglobin; PLT, platelet; CRP, C -reactive protein; PCT, procalcitonin; PT, Prothrombin time; APTT, activated partial thromboplastin time; FIB, fibrinogen; ALT, alanine aminotransferase; AST, aspartate aminotransferase; ALP, Alkaline Phosphatas; TBil, total bilirubin; TG, Triglycerides; TC, Total Cholesterol; sCr, serum creatinine; urea, blood urea nitrogen; CK-MB, Creatine kinase isoenzyme; TnIH, Troponin I High-sensitivity.

### Predicting fatal outcomes

**Figure 2:**
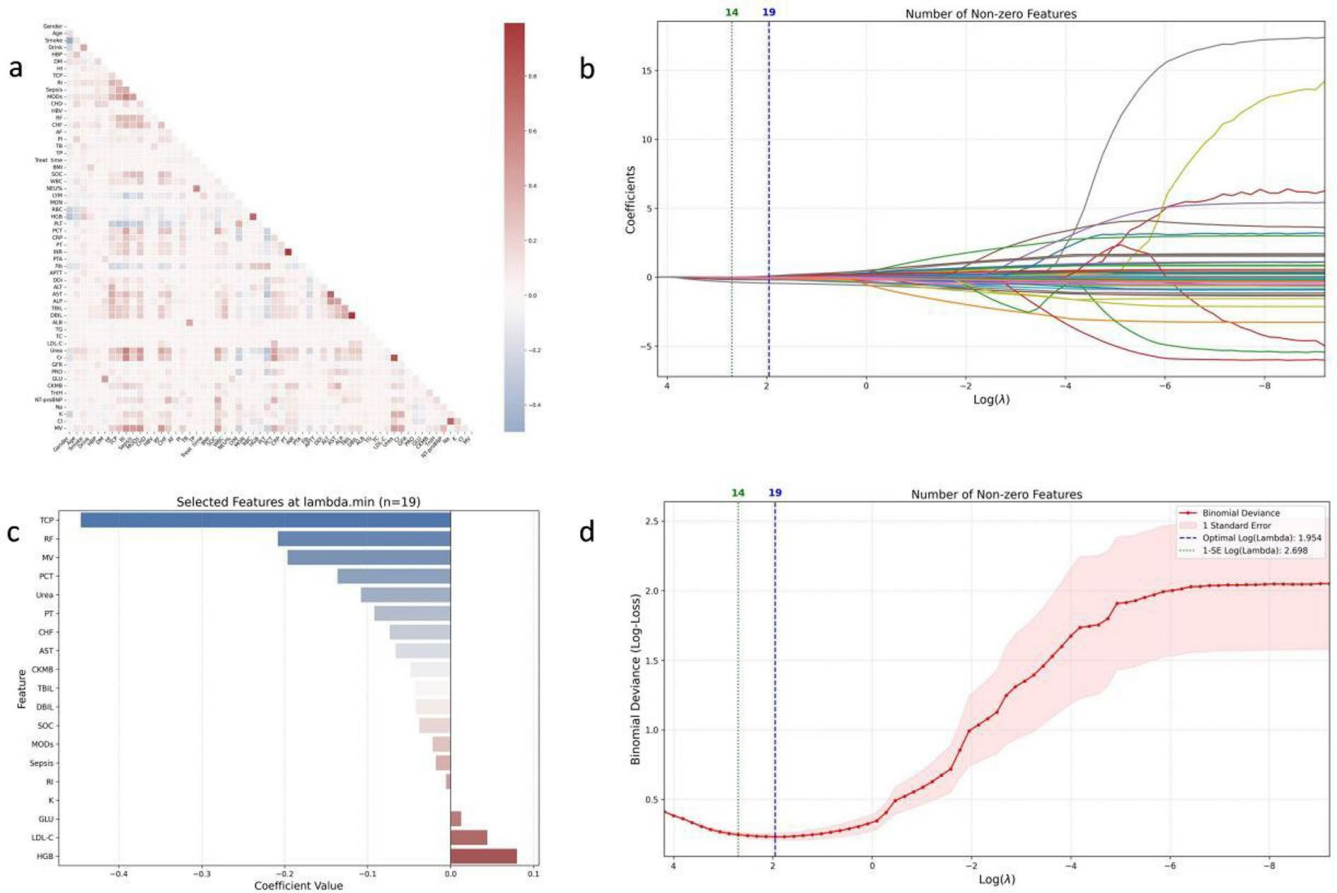
Flowchart of variable screening using logistic regression with L1 regularization based on mortality as the outcome. **(a)** Correlation heatmap of continuous variables, with larger pie charts indicating stronger correlations. **(b)** The coefficient profile plot was produced against the log (*λ*) sequence. The best penalty coefficient lambda was selected using a fivefold cross-validation and minimization criterion. **(c)** Coefficient bar chart of the 19 variables with non-zero coefficients selected by lambda.min. **(d)** Plot of binomial deviance versus log (λ) and a vertical dashed line, by validating the optimal parameter λ in the L1 regularization model. Nineteen variables with non-zero coefficients were selected using lambda.min.

This study used LASSO regression to analyze 56 candidate variables, ultimately selecting 19 predictors significantly associated with mortality outcomes, including: thrombocytopenia, respiratory failure, mechanical ventilation, PCT, Urea, PT, cardiac insufficiency, AST, CK-MB, TBIL, DBIL, altered mental status, MODS, sepsis, renal insufficiency, serum potassium (K), blood glucose, LDL-C, and HGB. The regression coefficients for each variable are shown in Figure 2c.

**Figure 3:**
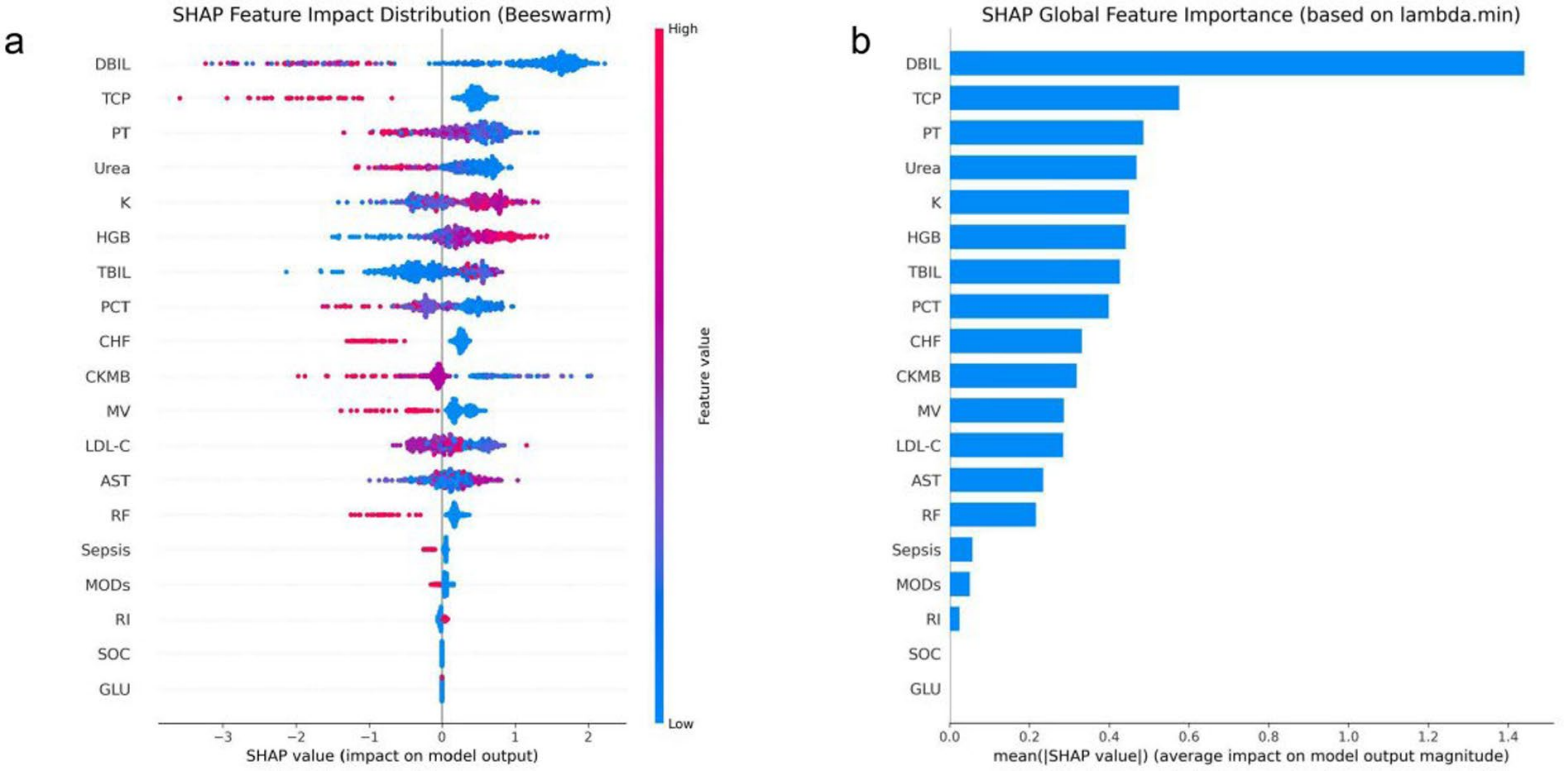
SHAP explanations of the mortality prediction model (XGBoost). **(a)** SHAP values of screened features, where each row represents a feature, the axis represents the SHAP value, red dots indicate higher feature values, and blue dots indicate lower feature values. **(b)** Variable importance ranking based on SHAP means.

**Figure 4:**
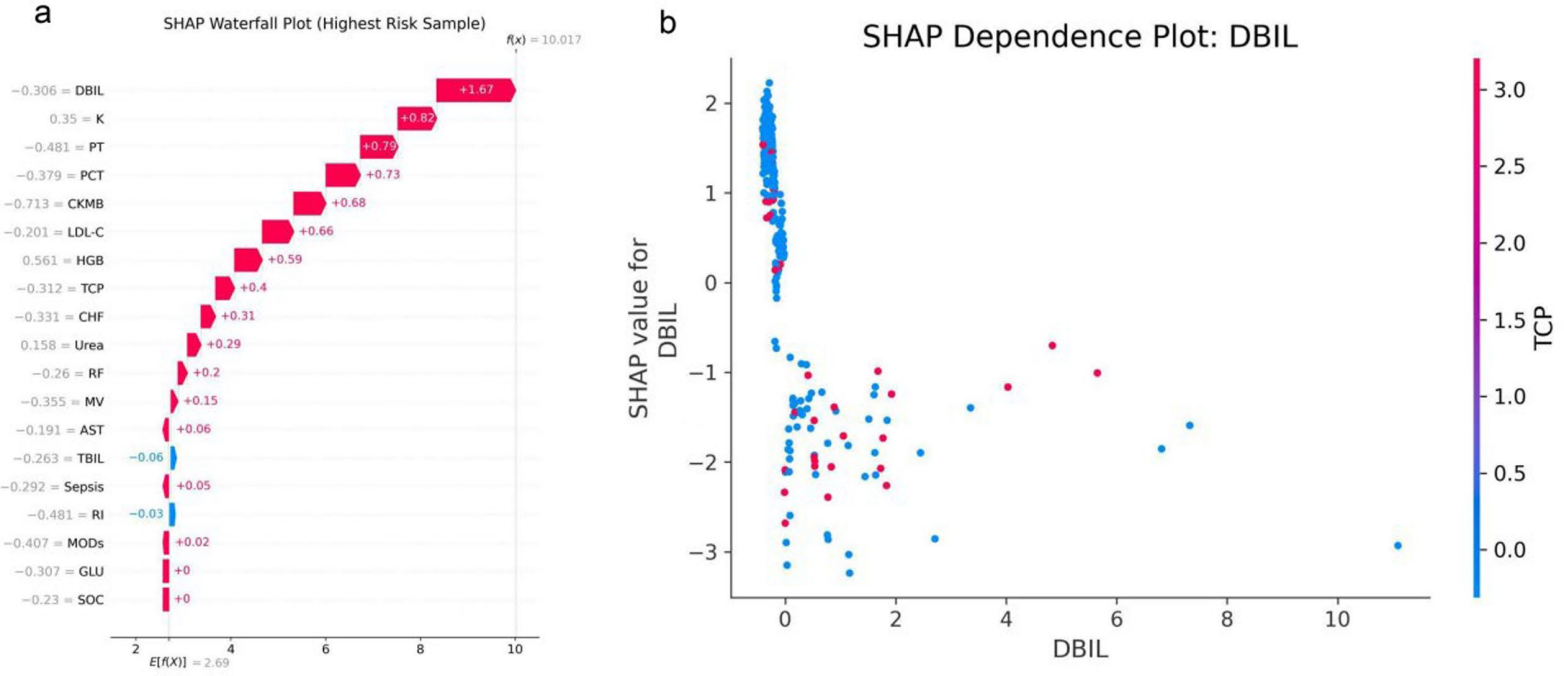
**(a)** SHAP predictions for severe scrub typhus outcome (XGBoost). Pink arrows indicate a positive impact of the feature on the prediction, while blue arrows indicate a negative impact. The length of the arrows reflects the magnitude of the impact, with longer arrows indicating a greater impact on the prediction. **(b)** The impact of DBIL on the prediction results of the XGBoost model.

Figure 3a’s SHAP visualization ranks the risk factors for predicting scrub typhus mortality, with DBIL, thrombocytopenia, PT, Urea, K, HGB, TBIL, and PCT being the most significant. Pearson correlation heatmap of candidate variables is shown in Figure 2a, and the cross-validation results of the regularization parameter are presented in Figures 2b,d. The intensity of red indicates higher risk values, while blue indicates lower risk values. Figure 3b displays the SHAP values for each feature from high to low. Figure 4a shows the contribution of each feature to the prediction, illustrating the interpretability of the model. Figure 4b illustrates the nonlinear influence of DBIL values on the prediction output of the XGBoost model.

### Predicting severe outcomes

**Figure 5:**
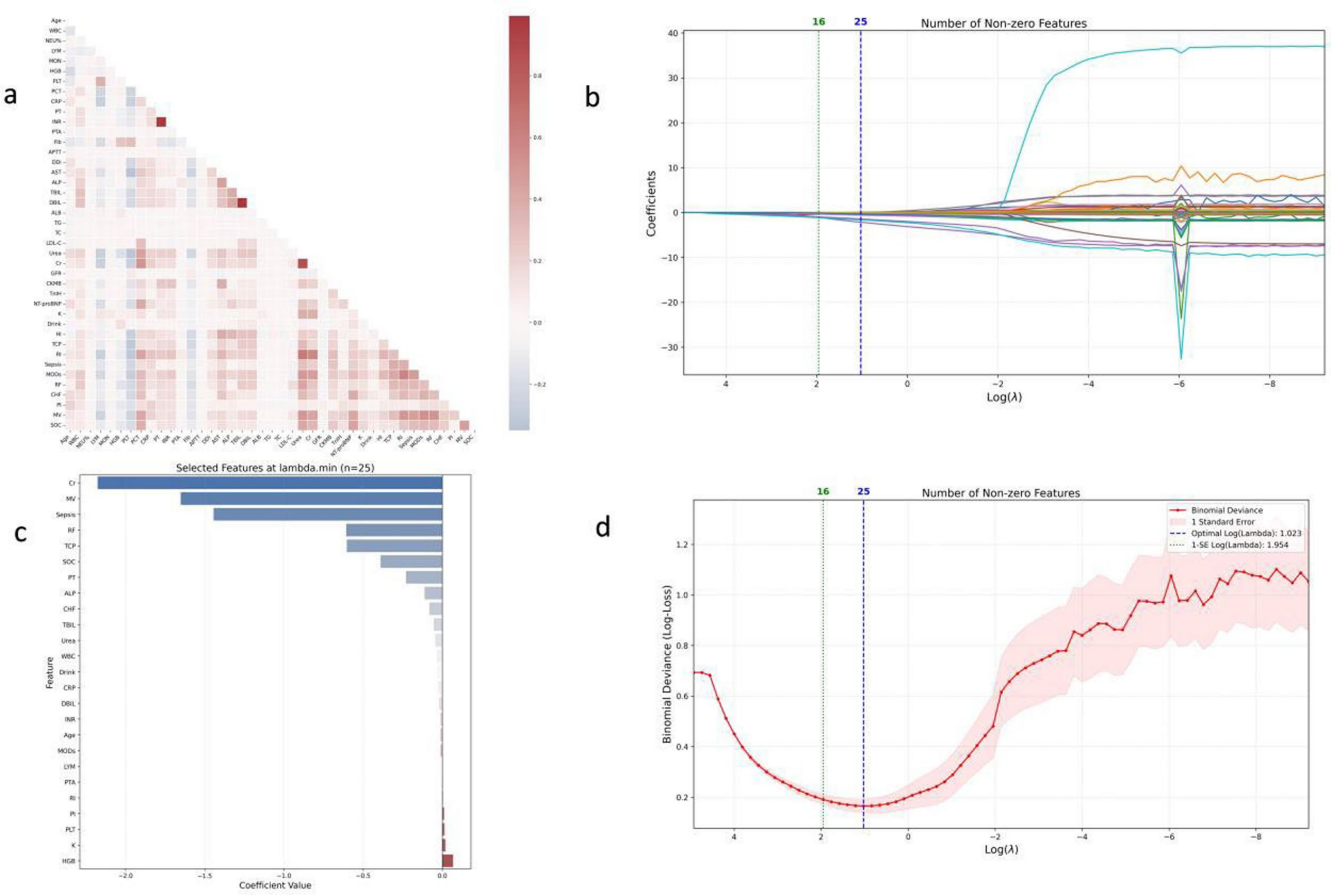
Variable selection process of logistic regression with LASSO regularization when the outcome is progression to severe scrub typhus. **(a)** Heatmap of included variables. **(b)** Five-fold cross-validation to determine the optimal regularization parameter λ. **(c)** 25 features selected by the LASSO regularized and their corresponding coefficients. **(d)** Dynamic paths of model coefficients with regularization strength (Log(λ)).

As shown in Figures 5a–d, to predict whether patients would progress to severe illness, variable selection was conducted using L1-regularized logistic regression, the same approach applied for mortality risk screening. A total of 25 features with non-zero regression coefficients were identified, including Cr, mechanical ventilation, Sepsis, RF, TCP, SOC, DBIL, WBC, INR, prothrombin time, fibrinogen, AST, MODS and hemoglobin. The coefficients of each selected variable are presented in Figure 5c.

**Figure 6:**
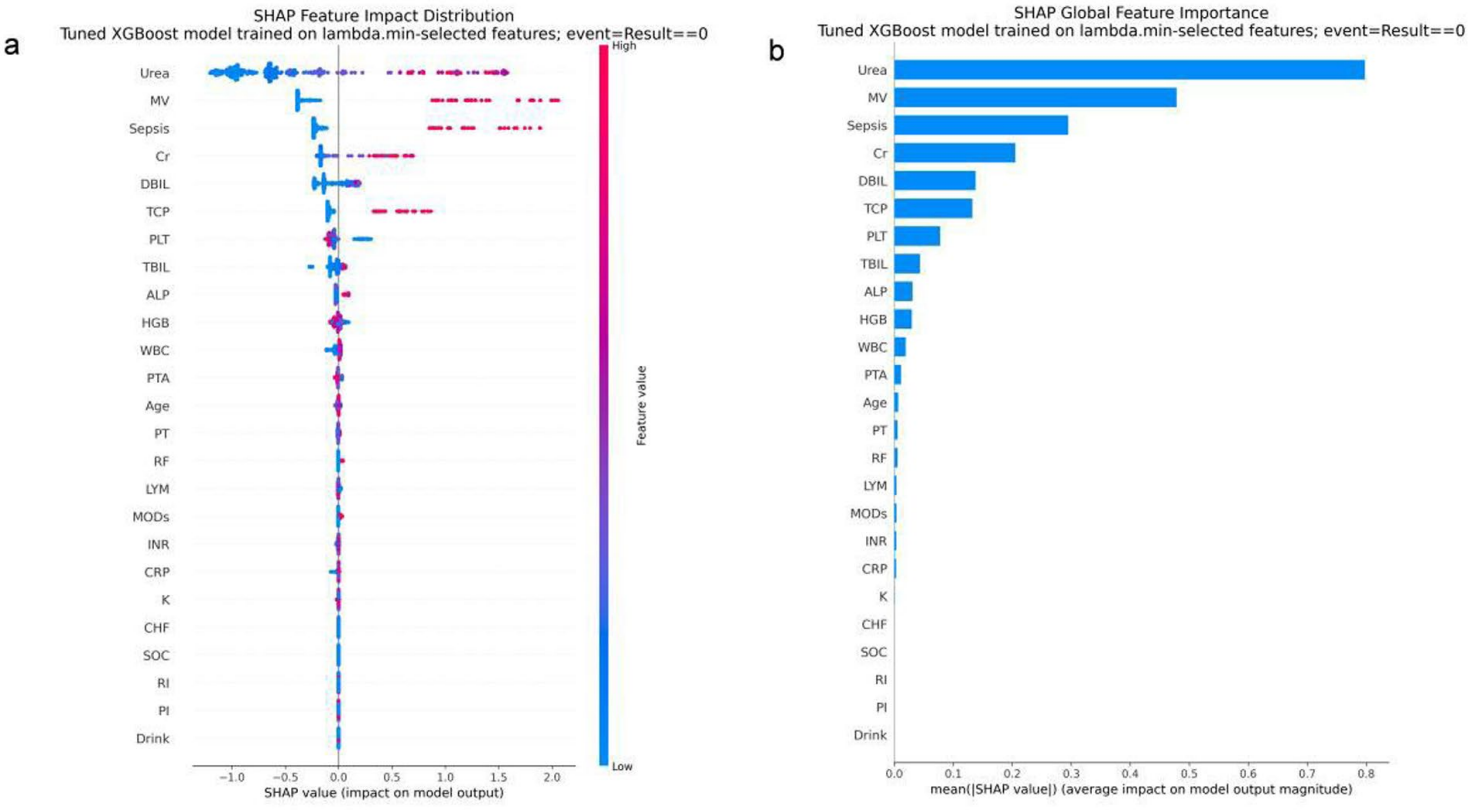
SHAP explanation of the severe prediction model (XGBoost). **(a)** SHAP values of screened features, each row represents a feature, the x-axis represents the SHAP value, red dots represent higher feature values, and blue dots represent lower feature values. **(b)** Variable importance ranking based on mean SHAP values.

Figure 6a ranks the risk factors for predicting progression to severe illness. The most influential factors include Urea, mechanical ventilation, Sepsis, Cr, DBIL, TCP, PLT, TBIL, ALP, hemoglobin and WBC. Red represents higher risk values, whereas blue indicates lower risk values. Figure 6b ranks all included features by mean SHAP value to demonstrate global feature importance.

**Figure 7:**
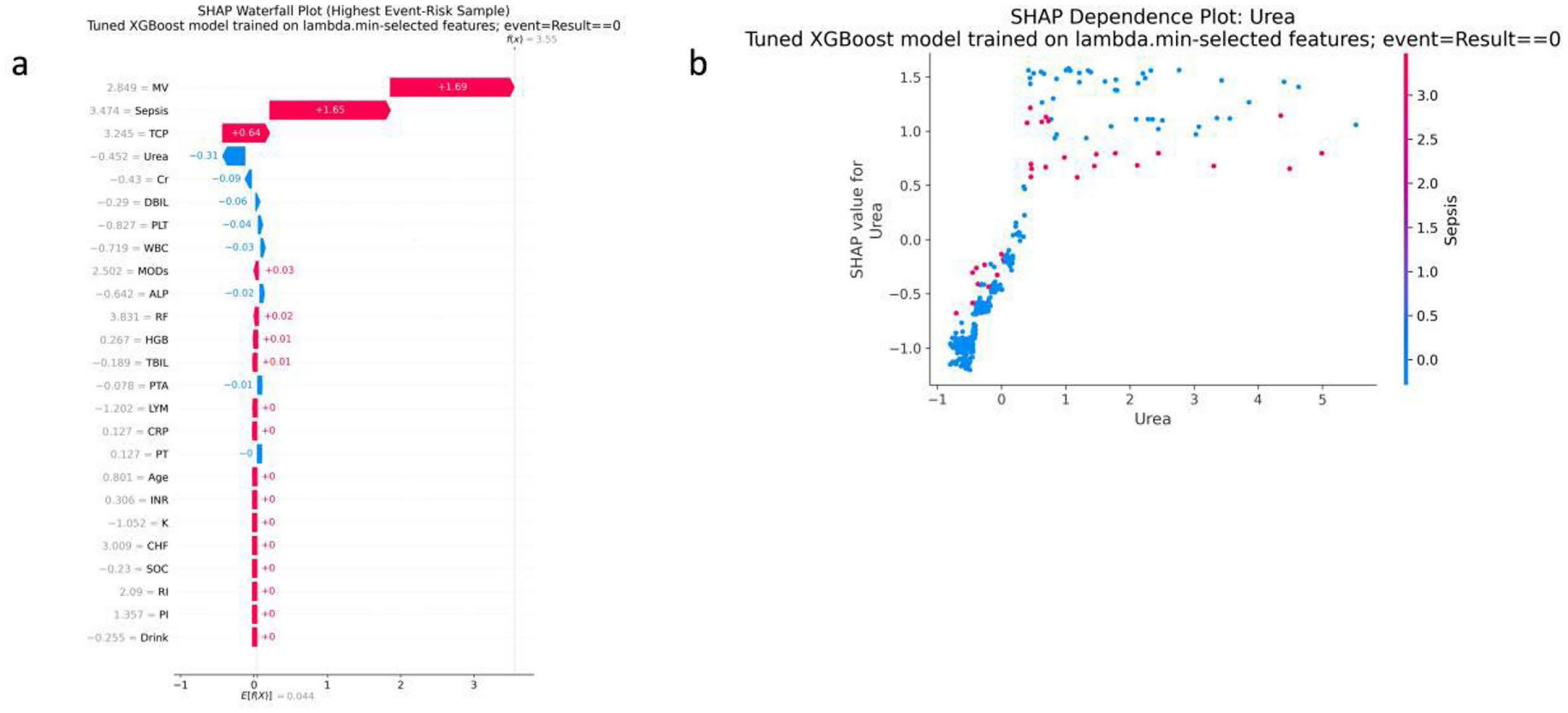
**(a)** SHAP predictions for severe scrub typhus outcome (XGBoost). Pink arrows indicate a positive impact of the feature on the prediction, while blue arrows indicate a negative impact. The length of the arrow reflects the magnitude of the impact, with longer arrows indicating a greater impact on the prediction. **(b)** The impact of Urea on the prediction results of the XGBoost model.

Figure 7a presents the contribution value of each feature for predicting progression to severe illness, thereby illustrating the interpretability of the model. Additionally, Figure 7b shows the impact of Urea, the most influential variable, on the prediction results of the XGBoost model. The higher the Urea level, the greater the probability of developing severe illness.

**Figure 8:**
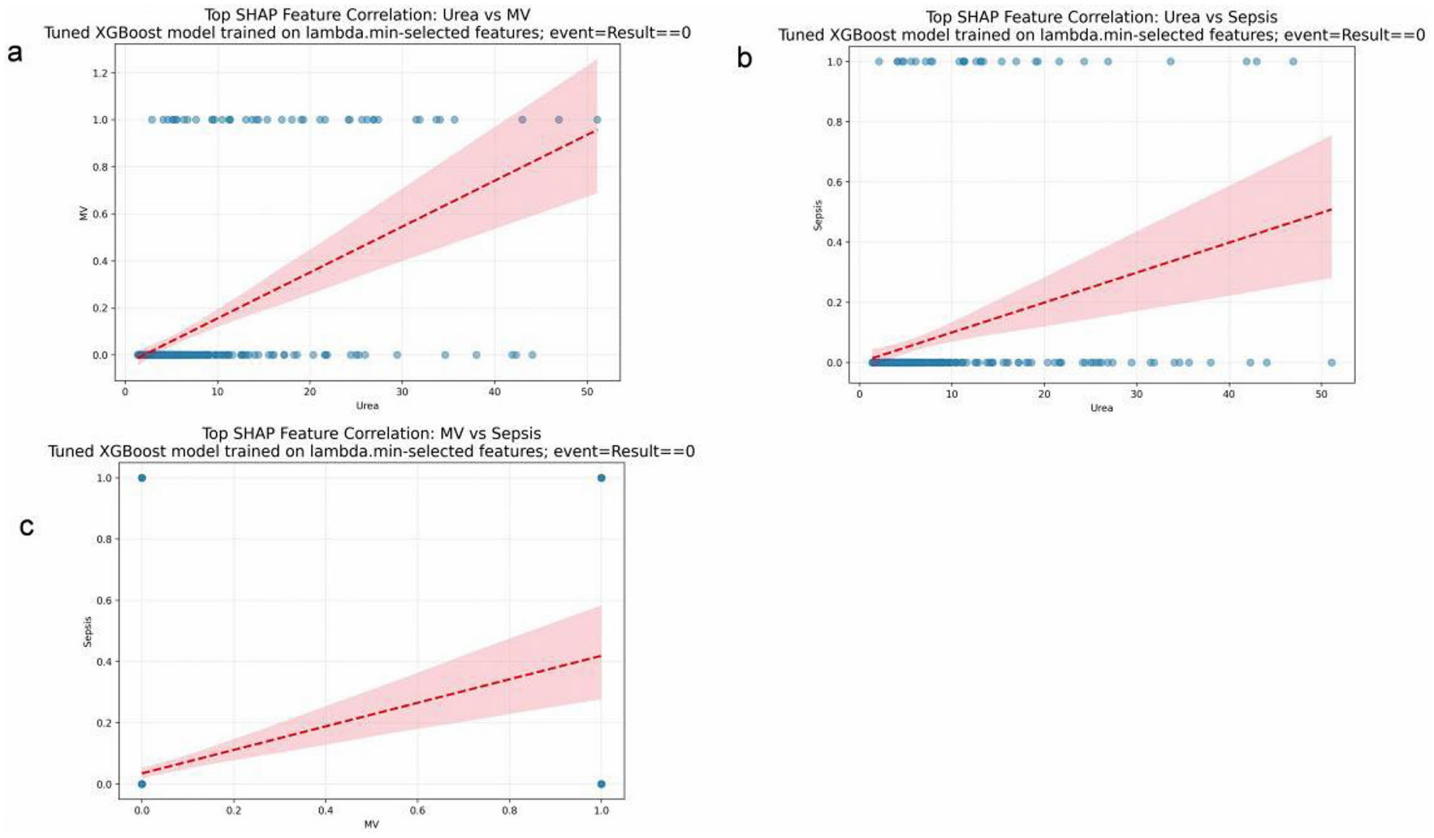
Dependency plots illustrating the interrelationships among the top three variables with the highest SHAP values. **(a)** Correlation between Urea and mechanical ventilation (MV). **(b)** Correlation between Urea and Sepsis. **(c)** Correlation between MV and Sepsis.

Figure 8 reveals the interrelationships among the three most important features — Urea, MV and Sepsis — for predicting severe outcomes in the XGBoost model. (a) Urea is positively correlated with MV. Patients with elevated urea levels tend to have more severe conditions and are more likely to require mechanical ventilation support. (b) Urea is positively correlated with Sepsis. The higher the urea level, the greater the probability of patients developing sepsis. (c) MV is positively correlated with Sepsis. Patients receiving mechanical ventilation have a higher prevalence of sepsis.

**Figure 9:**
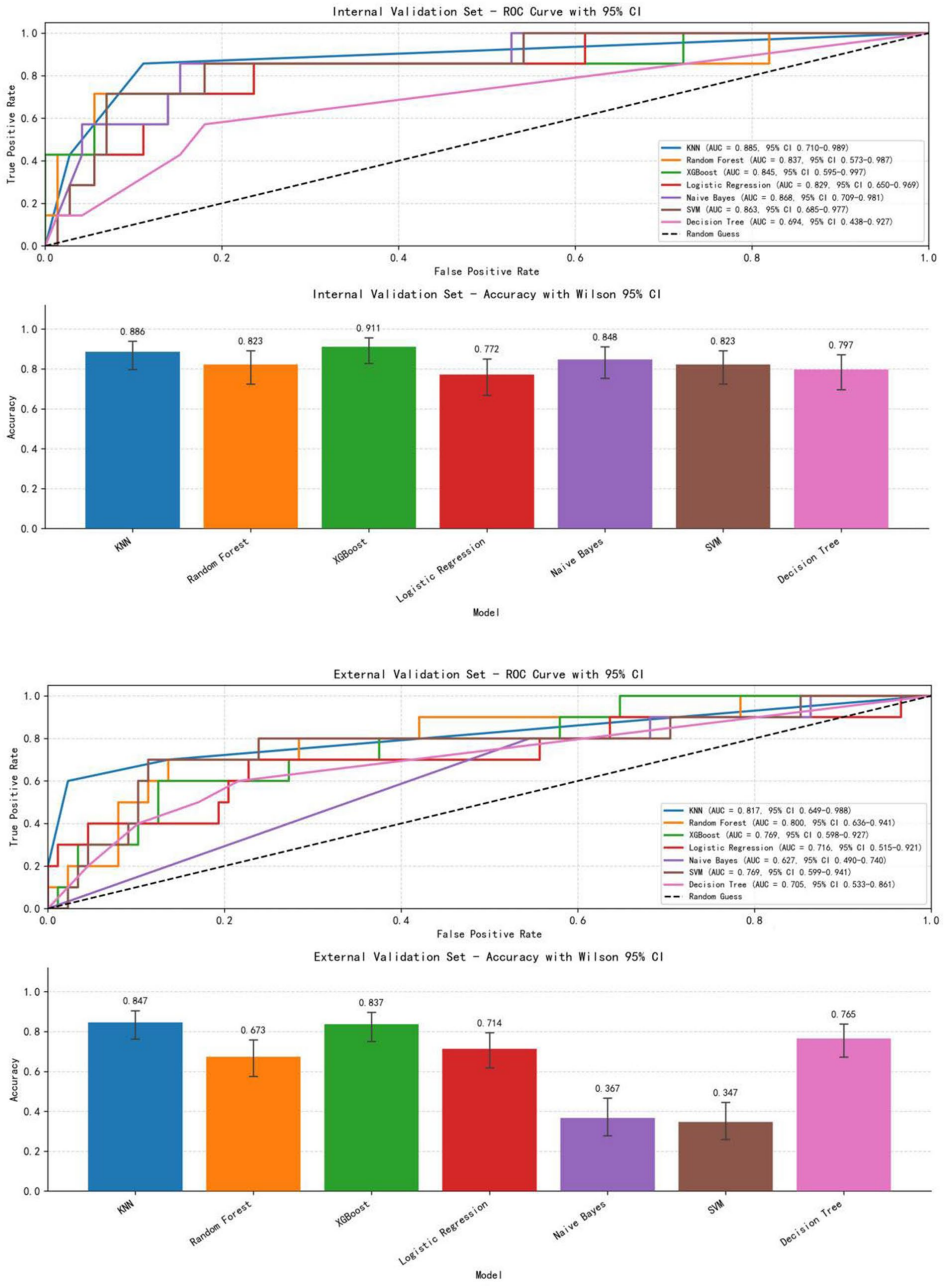
ROC curve areas of various models’ test sets and external validation sets in predicting mortality.

**Figure 10:**
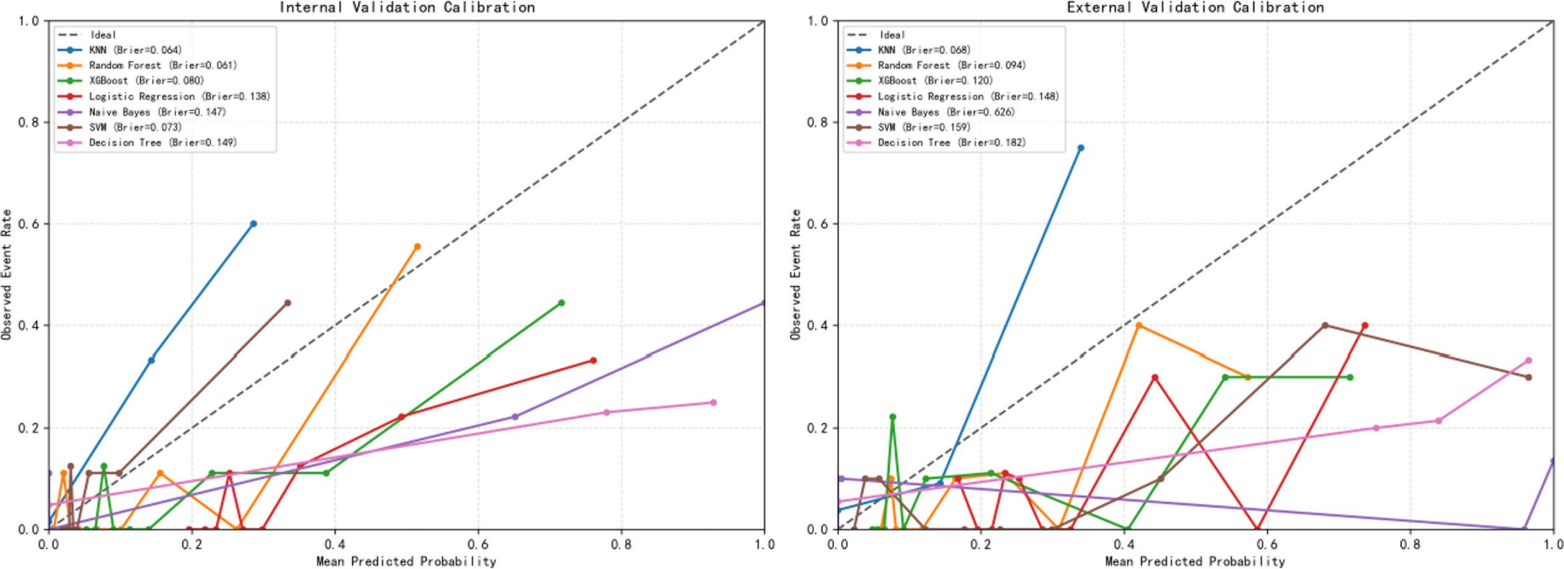
Calibration curves for each learning model predicting death.

**Figure 11:**
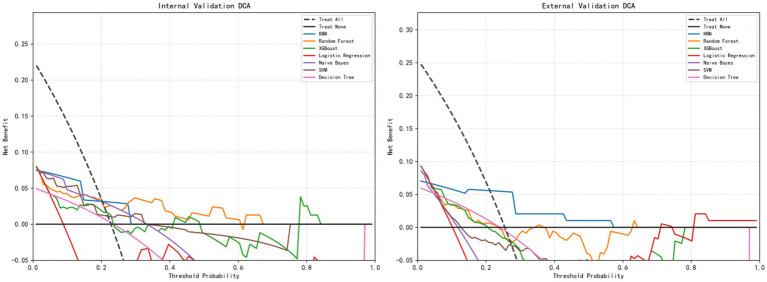
Decision curves for each learning model predicting death.

Among the seven prediction models compared for mortality prediction, as shown in Figure 9, most models performed well on the internal test set, with AUC values generally above 0.83. KNN (AUC = 0.885) and Naive Bayes (AUC = 0.868) achieved the best performance, followed by XGBoost (AUC = 0.845). All models had decreased AUC values on the external validation set, while KNN remained the most robust model (AUC = 0.817). Both KNN and XGBoost maintained high accuracy in internal and external validation. The comprehensive performance metrics of each model are listed in Table 2. All prediction models showed obvious calibration bias in external validation. Among all models, KNN exhibited the highest stability across internal and external validation and obtained the lowest Brier score (Figure 10). The results of Decision Curve Analysis (DCA) indicated that when the threshold probability ranged from 0.05 to 0.60, the net benefit of the KNN model was much higher than that of other models (Figure 11), suggesting that it has certain clinical application potential within this threshold range.

**Table 2:** Performance of 7 models in predicting mortality.

Model	AUC (95% CI)	Accuracy	F1	Sensitivity	Specificity	Brier score
KNN (train)	0.885 (0.710–0.989)	0.886	0.57	0.86	0.889	0.064
KNN (test)	0.817 (0.649–0.988)	0.847	0.48	0.70	0.864	0.068
RF (train)	0.837 (0.573–0.987)	0.823	0.46	0.86	0.819	0.061
RF (test)	0.800 (0.636–0.941)	0.673	0.33	0.80	0.659	0.094
XGboost (train)	0.845 (0.595–0.997)	0.911	0.63	0.71	0.944	0.080
XGboost (test)	0.769 (0.598–0.927)	0.837	0.43	0.60	0.864	0.120
LR (train)	0.829 (0.650–0.969)	0.772	0.40	0.86	0.764	0.138
LR (test)	0.716 (0.515–0.927)	0.714	0.33	0.70	0.716	0.148
NB (train)	0.868 (0.709–0.981)	0.848	0.50	0.86	0.847	0.147
NB (test)	0.627 (0.490–0.740)	0.367	0.21	0.80	0.318	0.626
SVM (train)	0.863 (0.685–0.977)	0.823	0.46	0.85	0.819	0.073
SVM (test)	0.769 (0.599–0.941)	0.347	0.20	0.80	0.295	0.159
Decision (train)	0.694 (0.438–0.927)	0.797	0.33	0.57	0.819	0.149
Decision (test)	0.705 (0.533–0.861)	0.765	0.34	0.60	0.784	0.182

AUC, Area Under the Roc Curve; F1, F1 score; KNN, K-Nearest Neighbors; RF, Random Forest; XGBoost, Extreme Gradient Boosting; LR, Logistic regression; NB, Naive bayes; SVM, Support Vector Machine; DT, Decision Tree.

**Figure 12:**
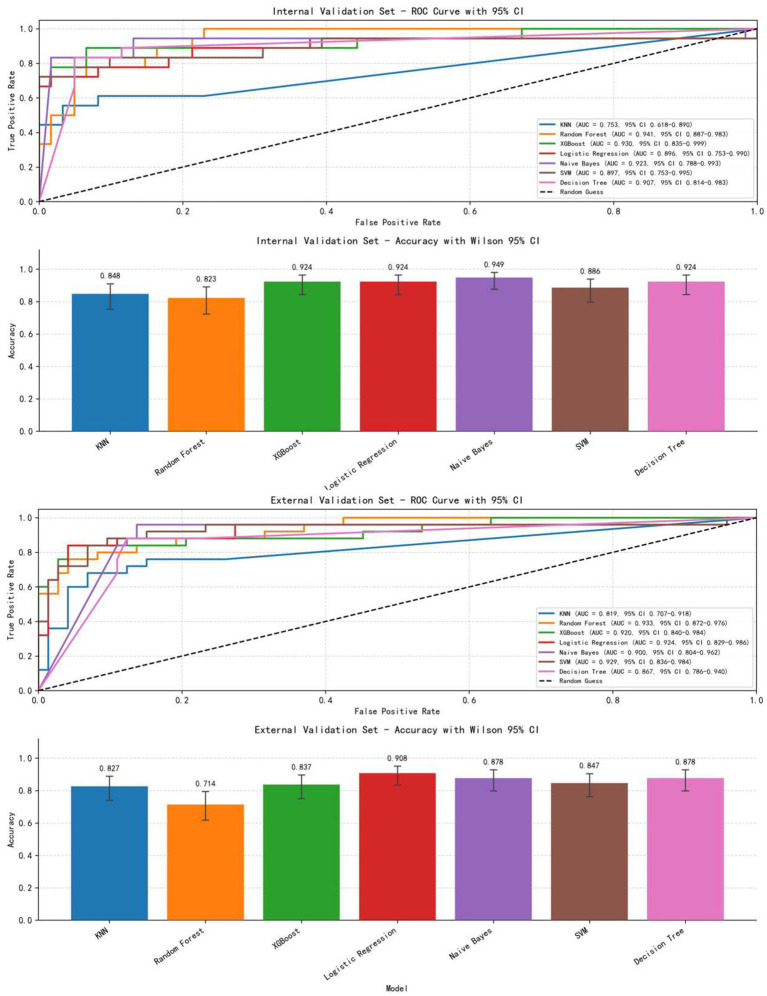
ROC curve area of the test set and external validation set for each model in predicting severe cases.

**Figure 13:**
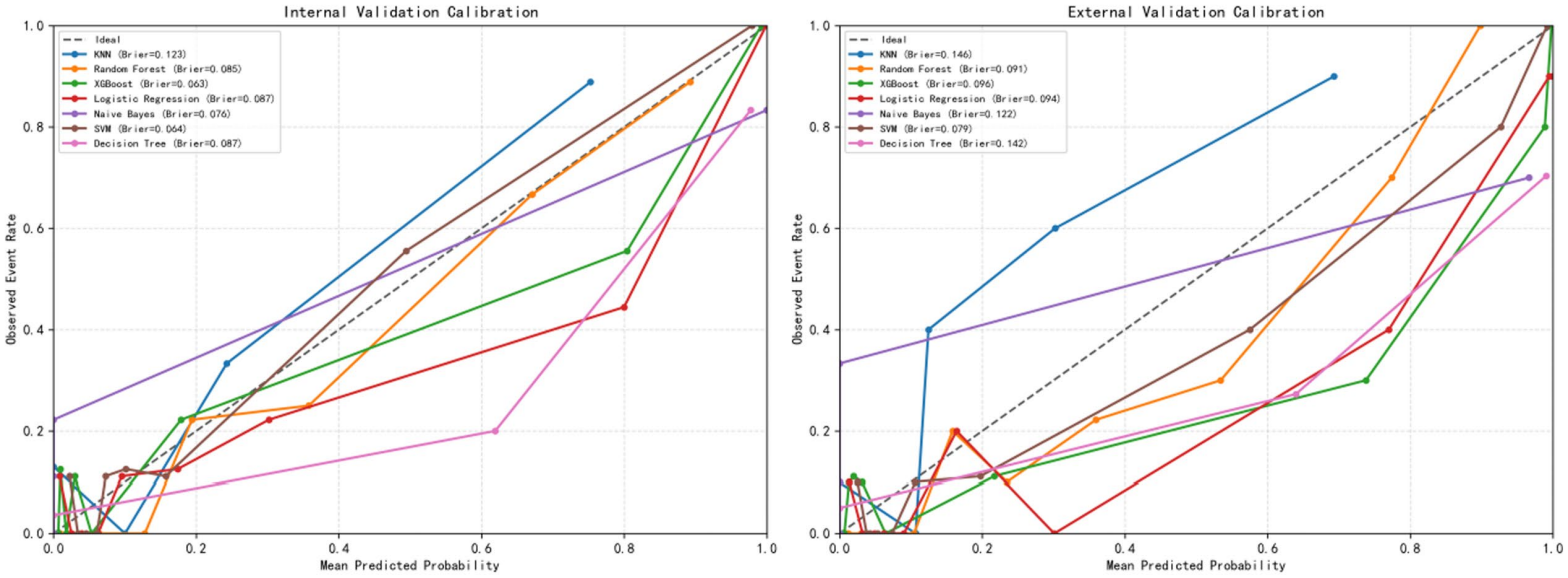
Calibration curves for each learning model predicting severe cases.

**Figure 14:**
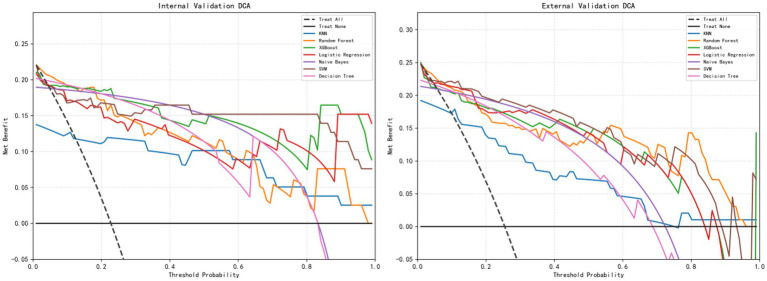
Decision curves of different machine learning models in predicting severe cases.

For the prediction of severe outcomes, 25 predictors significantly associated with progression to severe illness were selected from candidate variables using L1-regularized logistic regression. The corresponding regression coefficients are presented in Figure 5c. After incorporating these 25 variables into model construction, Figure 12 demonstrates that the Random Forest model delivered the optimal performance on both the internal training set and external validation set, with AUC values of 0.941 and 0.933, respectively. XGBoost ranked second, with AUC values of 0.930 and 0.920. Both models achieved high accuracy in the internal test set and external validation. Detailed performance metrics of each model are summarized in Table 3. According to the calibration curves (Figure 13), XGBoost and SVM showed the best calibration performance in both internal and external validation. DCA results revealed that when the threshold probability was between 0.05 and 0.80, SVM, Random Forest and logistic regression models all yielded positive net benefit in the training set and validation set (Figure 14), indicating that these three algorithms have high potential value for practical clinical risk assessment.

**Table 3:** Performance of 7 models in predicting severe *Scrub Typhus*.

Model	AUC (95% CI)	Accuracy	F1	Sensitivity	Specificity	Brier score
KNN (train)	0.753 (0.618–0.890)	0.848	0.65	0.61	0.918	0.123
KNN (test)	0.819 (0.707–0.918)	0.827	0.68	0.72	0.863	0.146
RF (train)	0.941 (0.887–0.983)	0.823	0.72	0.99	0.770	0.085
RF (test)	0.933 (0.872–0.976)	0.714	0.62	0.92	0.644	0.091
XGboost (train)	0.930 (0.835–0.999)	0.924	0.84	0.89	0.934	0.063
XGboost (test)	0.920 (0.840–0.984)	0.837	0.72	0.84	0.836	0.096
LR (train)	0.896 (0.753–0.990)	0.924	0.81	0.72	0.984	0.087
LR (test)	0.924 (0.829–0.986)	0.908	0.82	0.84	0.932	0.094
NB (train)	0.923 (0.788–0.993)	0.949	0.88	0.83	0.984	0.076
NB (test)	0.900 (0.804–0.962)	0.878	0.79	0.84	0.890	0.122
SVM (train)	0.897 (0.753–0.995)	0.886	0.77	0.83	0.902	0.064
SVM (test)	0.929 (0.836–0.984)	0.847	0.75	0.92	0.822	0.079
Decision (train)	0.907 (0.814–0.983)	0.924	0.83	0.83	0.951	0.087
Decision (test)	0.867 (0.786–0.940)	0.878	0.79	0.88	0.877	0.142

AUC, Area Under the Roc Curve; F1, F1 score; KNN, K-Nearest Neighbors; RF, Random Forest; XGBoost, Extreme Gradient Boosting; LR, Logistic regression; NB, Naive bayes; SVM, Support Vector Machine; DT, Decision Tree.

Overall, the key clinical and laboratory features screened by L1-regularized logistic regression can effectively support risk modeling for severe illness and mortality. For mortality prediction, KNN showed the strongest consistency and stability in terms of AUC, accuracy, calibration (Brier score) and clinical net benefit in both internal and external validation sets. In contrast, ensemble learning models including Random Forest and SVM achieved a superior balance between predictive performance and generalization ability for severe outcome prediction.

## Discussion

After *Orientia tsutsugamushi* invades the human body, it first proliferates at the bite site, and then spreads through the lymphatic and hematogenous systems, leading to rickettsiemia. This pathogen has a significant tropism for vascular endothelial cells, where it can parasitize and replicate intracellularly, causing systemic vasculitis and perivasculitis. This widespread vascular damage is the most fundamental pathological basis of scrub typhus, leading to increased vascular permeability, tissue congestion, edema, and potentially multi-site hemorrhage (e.g., gastrointestinal bleeding, pulmonary hemorrhage). Clinically, this manifests as varying degrees of organ damage and even organ failure, with common complications including severe pneumonia, acute kidney injury, and septic shock (Kim et al., 2019). Several studies have confirmed that the risk of death in patients with scrub typhus is closely related to organ failure, sepsis, and the need for mechanical ventilation support (Kim et al., 2010). Based on this, the present study further employed the LASSO regularization to perform feature selection with death and severe illness as outcome variables, respectively, ultimately identifying 19 predictors associated with death and 23 predictors associated with severe illness. A comparative analysis revealed 15 common features between the two sets (including AST, CKMB, MV, CHF, DBIL, HGB, MODS, PCT, PT, RF, RI, Sepsis, TCP, TBIL, Urea). These indicators collectively outline the progression pathway of severe scrub typhus: starting with intense inflammatory responses (PCT, Sepsis) and coagulation dysfunction (PT, TCP), further leading to multi-organ damage in the liver, kidneys, and heart (AST, TBIL, Urea, CHF, RI), and finally progressing to multiple organ dysfunction syndrome (MODS) and respiratory failure (RF, MV), systemically driving the disease towards a critical outcome.

This study employs SHAP feature importance maps to gain a robust, in-depth understanding of the clinical observations that certain factors are correlated with elevated mortality rates, and our proposed model effectively validates these critical clinical findings. The clinical phenotype observed in a prior clinical study — “respiratory failure is common, hepatic/coagulation failure is frequent and associated with high mortality” — has been quantified and stratified in the predictive model of this study (Pannu et al., 2021). In summary, the machine learning model for severe scrub typhus demonstrated that blood urea nitrogen (BUN, corresponding to the indicator Urea in this study) and mechanical ventilation (MV) were the most powerful predictive factors with the highest SHAP values, which directly validated that respiratory failure serves as the core clinical manifestation of severe scrub typhus. As a core prognostic biomarker for critically ill patients, BUN has been proven to be closely correlated with 28-day mortality, intensive care unit (ICU) stay duration, and multiple organ dysfunction (Huang et al., 2026; Deng et al., 2025). In the context of scrub typhus pathogenesis, systemic diffuse vascular endothelial injury and vasculitis caused by *Orientia tsutsugamushi* can induce microcirculatory disturbance and visceral hypoperfusion throughout the body. Meanwhile, severe inflammatory stress triggered by infection markedly accelerates systemic protein catabolism. The combined effects of the above pathological changes ultimately lead to elevated BUN levels in patients. Accordingly, the increase in BUN does not merely reflect renal impairment; it also indicates systemic hypercatabolism, severe inflammatory response, and generalized tissue hypoperfusion, which quantitatively mirrors the severity of systemic pathological damage in scrub typhus. Therefore, BUN elevation acts as a critical early warning sign of continuous disease progression and deterioration, which thoroughly explains its robust predictive value for severe outcomes and mortality in scrub typhus patients in this study.

Nevertheless, the model reveals deeper clinical and pathological implications, highlighting the fundamental role of hepatic and coagulation indicators in the prognostic prediction chain, as well as the critical early warning value of renal metabolic and perfusion abnormalities during disease deterioration. Direct bilirubin (DBIL) and prothrombin time (PT) ranked high in the mortality prediction model, indicating that hepatic and coagulation system injury acts as a core internal pathological driver of disease deterioration prior to or concurrent with overt respiratory failure. Notably, elevated BUN can occur earlier than dominant respiratory failure, reflecting early systemic inflammation, perfusion deficiency, and progressive multiple organ damage, thereby serving as a pre-emptive warning biomarker for deteriorating clinical status. In the severe outcome prediction model, DBIL ranked fifth in feature importance, while international normalized ratio (INR) and platelet count (TCP), which reflect severe coagulation disorders, also achieved high predictive weights. These findings clearly delineate the prognostic trajectory from severe illness to death in scrub typhus patients: progressive hepatic and coagulation failure constitutes the decisive factor affecting patient prognosis, whereas BUN-represented renal metabolic disorders and systemic hypoperfusion serve as vital auxiliary pathological features throughout the entire course of disease aggravation and fatal progression. Collectively, the machine learning models not only verify that hepatic failure is a powerful prognostic predictor but also demonstrate that combined hepatic and coagulation dysfunction forms the core pathological pathway leading to mortality. Furthermore, this study confirms that BUN-associated systemic stress and perfusion injury function as a crucial intermediate link connecting early disease deterioration and late-stage multiple organ failure (Valbuena and Walker, 2013; Lee et al., 2011). The previously proposed pathogenic mechanisms of “systemic vasculitis-like involvement” and “hepatic sinusoidal endothelial injury” provide a solid pathophysiological explanation for the above-mentioned model findings. The key severe predictive indicators screened by the machine learning model in this study, including total bilirubin (TBIL) and direct bilirubin (DBIL) reflecting hepatic dysfunction, as well as prothrombin time (PT), international normalized ratio (INR), and platelet count (TCP) representing coagulation disorders, together with renal and metabolic-related indicators verified in this study such as BUN and urea-to-creatinine ratio (UCR), are essentially specific biological manifestations of systemic endothelial injury induced by *Orientia tsutsugamushi* infection in the liver, circulatory system, and systemic metabolic and perfusion status.

Accordingly, based on the screened predictive features, we constructed predictive models for scrub typhus using multiple machine learning algorithms, with severe disease and death defined as the two binary outcome variables. The adopted models included logistic regression, support vector machine, random forest, extreme gradient boosting (XGBoost), naive Bayes, and k-nearest neighbor (KNN). For severe outcome prediction, most models achieved stable and favorable predictive performance, with the area under the curve (AUC) values of ensemble models including random forest and XGBoost exceeding 0.8, indicating reliable discriminatory power for identifying severe scrub typhus. Decision curve analysis (DCA) further verified the favorable clinical applicability of the random forest and XGBoost models. In external validation, XGBoost and random forest exhibited outstanding generalization performance, with AUC values of 0.8878 and 0.9286, respectively. Such superior performance can be attributed to the capability of ensemble learning algorithms to effectively capture complex nonlinear relationships and interaction effects among clinical variables. Meanwhile, the built-in regularization and resampling mechanisms of these algorithms enhance model generalization ability and mitigate overfitting. These findings further demonstrate that the prediction model constructed based on core clinical biomarkers possesses robust generalization performance and promising clinical translation value for the early identification of severe scrub typhus.

Compared with severe outcome prediction, all models yielded slightly inferior performance in mortality prediction, which may be attributable to the limited number of fatal cases in the current cohort. Specifically, only 36 death cases were included in the internal validation set and merely 10 in the external validation set, which inevitably restricted the stability and generalizability of mortality prediction models. The naive Bayes model showed a favorable AUC of 0.86 in the training set but a substantial decline to 0.718 in the external validation set, suggesting obvious overfitting. In contrast, XGBoost presented consistent and stable performance across training and validation sets, with AUC values of 0.856 and 0.814, respectively. DCA results indicated that only the KNN model yielded positive net clinical benefit within the threshold probability range of 0.30–0.95 in both internal and external validation sets, albeit with limited overall discriminatory ability. After comprehensive evaluation of multiple predictive metrics including sensitivity, specificity, F1-score, and AUC, random forest and XGBoost were identified as the optimal prediction models.

Notably, XGBoost achieved excellent generalizability in the prediction of both severe disease and mortality outcomes, which can be explained by multiple inherent algorithmic advantages. First, XGBoost is well-suited to capture the complex nonlinear correlations prevalent in real-world clinical data. Second, it allows adaptive weight adjustment for class-imbalanced datasets, effectively improving the identification ability for minority clinical outcomes such as death and severe illness. Third, the built-in regularization mechanism further suppresses model overfitting after feature selection. Additionally, the model can output quantitative feature importance, facilitating intuitive and credible clinical interpretation of predictive results. Therefore, XGBoost is particularly applicable to clinical binary prognostic prediction scenarios characterized by a moderate sample size, appropriate feature dimensionality, and imbalanced outcome distribution, which perfectly matches the research setting of the present study. In comparison, KNN is susceptible to the curse of dimensionality when processing high-dimensional clinical features. The naive Bayes algorithm relies on the assumption of feature independence, which is rarely satisfied in real clinical datasets. Conventional decision trees suffer from unstable structure and high overfitting risk. These inherent limitations collectively compromised the predictive performance of the above models in this study.

In recent years, machine learning has been increasingly applied to the prediction of infectious disease prognosis. Taking scrub typhus as an example, a study in Fujian was the first to attempt using machine learning methods to predict disease severity (Lu et al., 2025). The constructed random forest model achieved a sensitivity of 98.3%, specificity of 96.6%, and an AUC of 0.981, demonstrating excellent classification performance. However, the study did not conduct external validation, and the model’s generalization ability remains to be confirmed. Furthermore, it failed to analyze mortality outcomes, limiting its comprehensive application in clinical risk stratification. Building upon this foundation, the present study integrates data from three medical institutions for training and validation, not only exhibiting excellent and generalizable performance in predicting severe outcomes but also further extending to predict mortality outcomes, significantly enhancing the model’s practical value in real clinical scenarios. Especially in primary healthcare institutions, where diagnostic and treatment equipment and professional human resources are relatively limited, this model relies on routinely accessible laboratory indicators such as direct bilirubin, prothrombin time, and platelet count. Combined with explainable artificial intelligence techniques, it can effectively identify patients at risk of developing severe disease early in the clinical course, providing a generalizable assisted decision-making pathway for primary care units to implement timely and precise interventions. Nonetheless, this study still has several limitations. First, although data from three centers were included, the total sample size remains limited, especially with a small number of cases for the mortality outcome, which may affect the stability and generalizability of the models, particularly complex ones. Second, all data were from institutions within Guangdong Province, and the population characteristics and diagnostic and treatment standards are relatively homogeneous, so the performance of the models in other epidemic areas still requires further external validation. Furthermore, although this study shows good predictive potential, its actual clinical utility still needs to be verified through prospective studies.

## Conclusion

In conclusion, this study demonstrated that artificial intelligence models based on routine admission indicators can effectively stratify disease severity and achieve promising prognostic prediction for patients with scrub typhus. Random forest and XGBoost were identified as the optimal predictive models. Among them, XGBoost exhibited stable performance in both the training and external validation sets for the prediction of severe illness and mortality, making it suitable for developing an early severe case identification tool for primary hospitals. Leveraging readily available clinical indicators, including blood urea nitrogen, direct bilirubin, prothrombin time, and platelet count, combined with explainable artificial intelligence approaches, this tool can effectively screen high-risk patients and provide a critical evidence basis for rapid triage and precise clinical referral in scrub typhus management.

### Limitations

This study has several inherent limitations. First, although clinical data were collected from three medical centers, the overall sample size was still relatively small. In particular, the number of fatal cases was limited, which may impair the stability and generalizability of the established models, especially complex algorithms. Second, this was a retrospective study based on electronic medical records, inevitably leading to missing data and recording bias. Furthermore, all enrolled participants were recruited from medical facilities within Guangdong Province. The homogeneity of population characteristics and clinical diagnostic and treatment standards restricts the extrapolation of our findings. Further external validation is required to confirm model performance in other endemic areas. Lastly, despite the promising predictive performance observed in this research, its practical clinical value needs to be verified in prospective studies.

## Data Availability

The raw data supporting the conclusions of this article will be made available by the authors, without undue reservation.
